# Timing matters: evaluating lateral spreads response disappearance as a prognostic marker in microvascular decompression for hemifacial spasm: a phenomenological study

**DOI:** 10.1007/s00701-025-06642-0

**Published:** 2025-08-29

**Authors:** Ahmed Al Menabbawy, Marie Eisold, Ehab El Refaee, Ina Lange, Ines Peters, Marc Matthes, W. S. Schroeder

**Affiliations:** 1 Department of Neurosurgery, University Medicine Greifswald, Greifswald, Germany; 2 Department of Neurosurgery, Cairo University, Cairo, Egypt

**Keywords:** Endoscope assissted, Hemifacial spasm, Lateral Spread response, Microvascular decompression, Neuromonitoring, Prognosis, Timing of LSR disappearance

## Abstract

**Purpose:**

Prognostic significance of lateral spreads response (LSR) disappearance in microvascular decompression (MVD) for hemifacial spasm (HFS) remains controversial. Still the timing of LSR disappearance and its association with overall outcomes has not been sufficiently investigated. We evaluate the prognostic significance of the timing of LSR disappearance during MVD in HFS.

**Methods:**

Prospective documentation of the LSR-Status during the procedural steps was performed alongside routinely collected data. Surgical steps were categorized into three phases: Opening phase (skin incision till cisternal opening), arachnoid dissection, and actual Decompression phase. Outcome assessment was conducted after a follow-up period of at least 12 months, with favorable outcome defined as at least 90% resolution of the spasms.

**Results:**

214 patients were included with a mean age (SD) of 54.9 ± 11.6 years and a follow-up duration (SD) of 25.8 ± 15.7 months. The male-to-female ratio was 1:1.6. LSR was "not detected" in 32 patients (15.0%), with a 93.8% favorable outcome. LSR "persisted" in 22 patients (10.3%), showing only 77.3% favorable outcome. In 16 patients (7.4%), LSR disappeared during the opening phase, yielding a 100% favorable outcome. LSR disappearance occurred during arachnoid dissection in 40 patients (18.7%), with a 91.1% favorable outcome. Finally, LSR disappearance following nerve decompression was observed in 104 patients (48.6%), showing a 78.9% favorable outcome. Earlier disappearance of the LSR was associated with long-term cure (P-value < 0.05).

**Conclusion:**

LSR may serve as a valuable intraoperative indicator during MVD for HFS. Early intraoperative disappearance of the LSR may predict favorable long-term outcomes. However, the disappearance of the LSR in general does not consistently correlate with surgical success.

## Introduction

The lateral spread response (LSR) is an abnormal electrophysiological phenomenon commonly observed in patients with hemifacial spasm (HFS) [8, 16]. It occurs when stimulation of one branch of the facial nerve elicits responses not only in the muscles innervated by the stimulated branch but also in additional muscle groups innervated by other branches of the facial nerve. Notably, this aberrant response often resolves following adequate decompression of the facial nerve [6].

However, the prognostic significance of the LSR in microvascular decompression (MVD) for HFS has been a subject of ongoing debate [8, 9, 30]. While some studies suggest that the resolution of LSR correlates with favorable short-term outcomes following MVD, its long-term prognostic value remains uncertain. Moreover, only a limited number of studies have addressed the clinical importance of the timing of LSR disappearance during different phases of MVD surgery [4, 16, 26].

Therefore, the objective of our study is to investigate the significance of the timing of LSR resolution during various stages of MVD in patients with HFS on both the short-term and long-term outcomes in our patients´ series.

## Materials and methods

### Study design

This study was designed as a prospective controlled cohort investigation with meticulous, prospective documentation of the intraoperative LSR conducted by the neurophysiology team. LSR data were collected preoperatively and during defined procedural stages of the MVD surgery to ensure comprehensive monitoring until the procedure is finally concluded. The study design and reporting were conducted in accordance with the Strengthening the Reporting of Observational Studies in Epidemiology (STROBE) guidelines. Local institutional ethics committee approval and informed written participants consent were obtained preoperatively.

The surgical procedure was systematically categorized into three distinct phases to facilitate structured and objective evaluation. The first procedural phase, termed the"opening phase,"included the skin incision, craniotomy, and the opening of the dura and cisterns with CSF release. The second phase focused on arachnoid dissection “Dissection phase”, a critical step to expose the neurovascular structures while minimizing nerve irritation. The third and final phase “Decompression phase” involved active nerve decompression using Teflon felt to separate the offending vessel from the nerve REZ. Patients with persistent LSR at the end of the operation were also recorded. Some patients did not have LSR before the operation and these patients were considered our control group when comparing with the other groups.

### Inclusion and exclusion criteria

Patients who underwent surgery between January 2017 and June 2023 were included in the study. Only those with a minimum follow-up period of at least twelve months were considered eligible. Patients with unreliable or unclear LSR curves were excluded.

### Electrodes placement and surgical technique

**Fig. 1:**
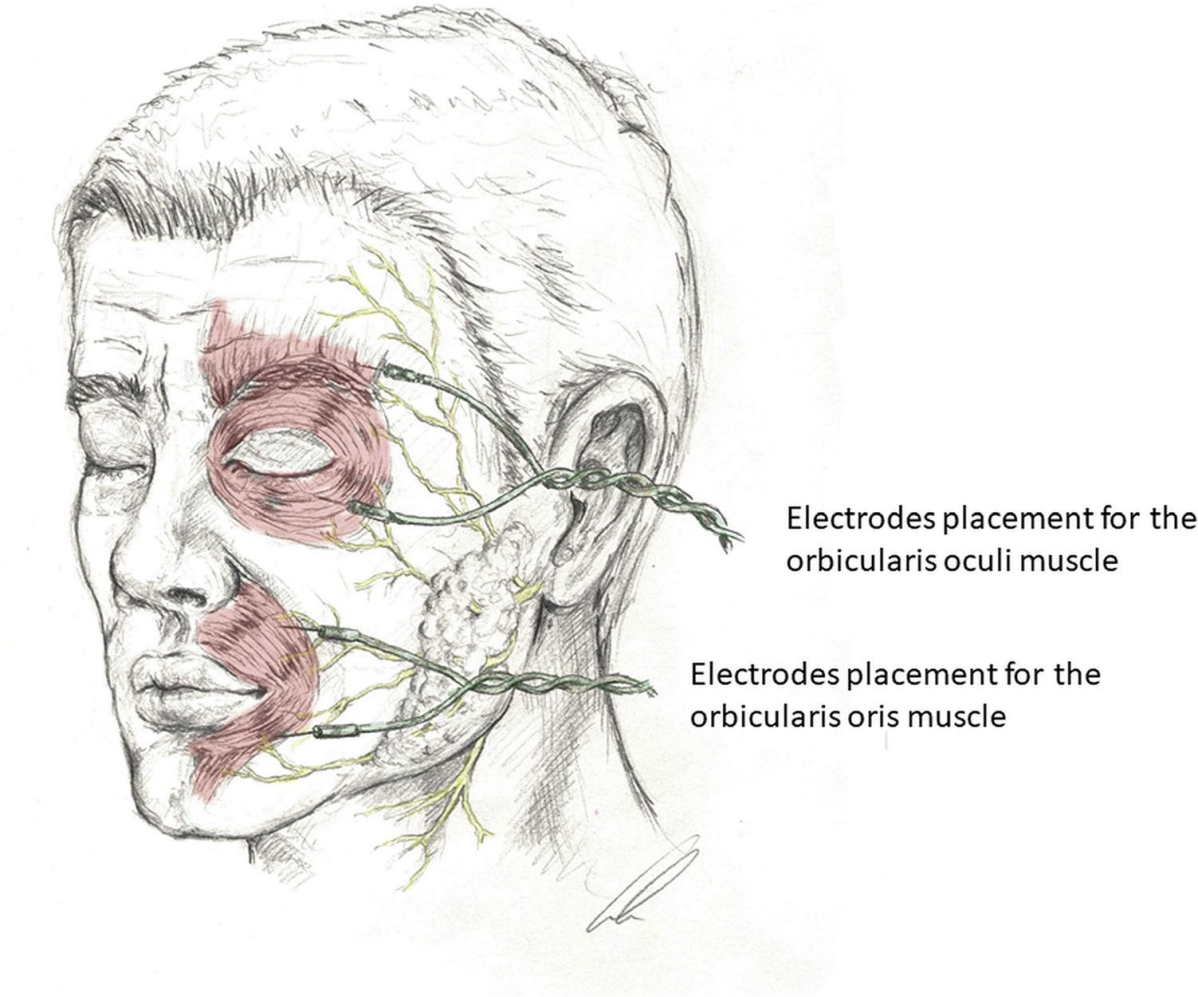
Illustrates the placement of electrodes on the orbicularis oculi and orbicularis oris muscles. Two needle electrodes are inserted into each muscle—one serving as a stimulator and the other recording free-running electromyographic (EMG) activity. A lateral spread response is observed when stimulation of one muscle elicits an abnormal response in the other muscle

All MVD procedures were carried out by the senior surgeon (H.W.S.S.). The procedures were performed with the patient in the supine position under continuous neurophysiological monitoring using facial electromyography and brainstem auditory evoked potentials (BAEP). LSR was systematically recorded during the procedure from both the orbicularis oculi and orbicularis oris muscles as shown in Fig. 1. A pulse wavelength of 0.2-ms with an intensity of 5 to 20 mA was used. We tried to use the least possible amplitude capable of producing evident LSRs as higher intensities may elicit an artefact like LSRs and resulting in false results [3]. In most of the patients, the values were around 10 mA. The LSR were recorded all over the operation time during evoked facial EMG monitoring that appeared in the other facial muscles, including the orbicularis oculi and the orbicularis oris, when the nerve that mainly innervates the frontalis muscle was stimulated. When the LSR was found to be persistent despite decompression, we explored further endoscopically all around the facial nerve and especially at the root exit zone (REZ) for any suspicious underlying structure that could represent residual compression [8]. Regarding BAEP, both ears were stimulated independently, using alternating rarefaction and condensation clicks with at least a 95-dB hearing level. A stimulus rate of 11.1 Hz was used [8, 9]. Working with adequately experienced neuromonitoring team is crucial to avoid false positive and false negative LSR.

Total Intravenous Anesthesia (TIVA) with avoidance of intermediate-acting non-depolarizing muscle relaxants (NDMRs) was always performed. Additionally, nimodipine and dexamethasone were administered intravenously.

After having performed a lower retrosigmoid craniotomy and opening of the dura, the basal cistern was opened under microscopic view. This step relaxed the cerebellum, and no spatula retraction was needed for the decompression [1, 2]. Then, sharp arachnoid dissection between lower cranial nerves and flocculus was performed to access the REZ of the facial nerve at the pontomedullary sulcus [14, 25]. Once the arachnoid dissection was complete, the REZ of the facial nerve was meticulously examined with the aid of a 45° endoscope to enhance visualization and ensure precise assessment [10, 29]. The course of the facial nerve was routinely inspected, and decompression was tailored to the anatomical findings. Depending on the specific anatomy, decompression was achieved using shredded Teflon, Teflon bridge or a Teflon/Gore-Tex sling secured to the skull base dura. The primary goal of the decompression was to eliminate any direct contact between the facial nerve and surrounding blood vessels, arachnoid, or even the Teflon material. While Teflon can be used for interposition, every effort was done to prevent direct contact between the facial nerve and the Teflon. The duration of the procedure varied between 100 and 240 min, depending on the complexity of the vascular compression.

### Variables and outcome parameters

Spasm improvement was assessed by patients using a 100-point scale. Evaluations were conducted immediately after surgery, at hospital discharge, at 3, 6, and 12 months, and then annually thereafter. Surgical outcomes were categorized into four grades: (1) *Excellent*, indicating complete resolution (100% cure); (2) *Very Good*, reflecting more than 90% improvement; (3) *Good*, representing 50%–90% improvement; and (4) *Poor*, with less than 50% improvement.

The incidence of complications was documented, with particular attention given to facial nerve involvement, which was analyzed separately. Key demographic parameters, including age, sex, duration of symptoms since diagnosis, and follow-up duration, were also recorded. These variables, along with the outcome measures, served as points of comparison among the different patient groups.

## Statistical methods

Statistical analyses were conducted using Social Science Statistics [27]. Continuous variables were summarized using mean ± standard deviation, while categorical variables were presented as counts and percentages. Comparative analyses between groups were performed using the Chi-square test and Fisher's exact test for categorical data, and ANOVA for continuous variables.

All predictors were selected a priori. A p-value ≤ 0.05 (two-tailed) was considered statistically significant.

## Results

### Demographics

**Fig. 2:**
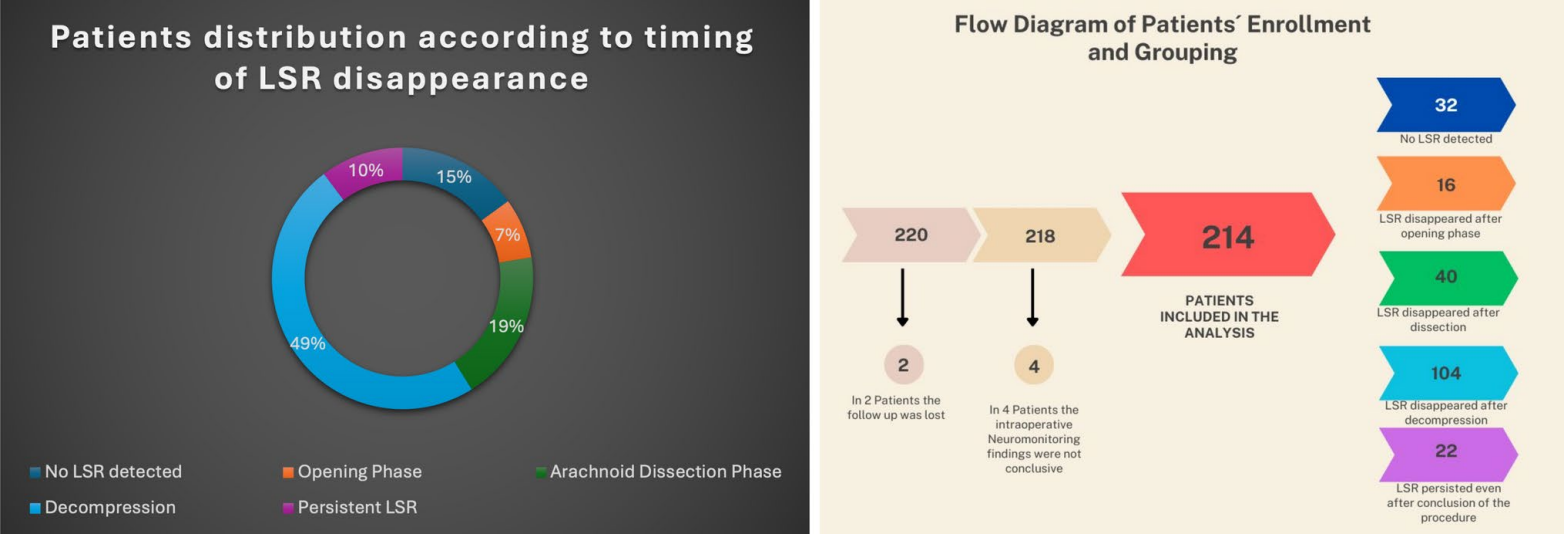
The right panel presents a flow diagram detailing the selection process of the 214 patients included in the study, as well as their subsequent allocation into groups based on the timing of the disappearance of the lateral spread response. Left: Showing a pie chart of the distribution of the patients among the different groups

220 Patients were initially enrolled in the study. Follow-up was lost for two patients and in four patients the intraoperative neuromonitoring results were not conclusive. Ultimately, 214 patients were completely included at the end and analyzed as shown in Fig. 2. The mean age (SD) averaged 54.9 ± 11.6 years, and the average follow-up duration (SD) is 25.0 ± 15.6 months. The male-to-female ratio was 1:1.58. 191/219 Patients improved following the MVD (89.5%). 16 patients (7.3%) experienced postoperative facial palsy which persisted only in 3 patients (1.4%).

### Classification according to LSR

The patients were classified according to presence or persistence of the LSR into five different patient groups (Fig. 2). LSR were not detected in 32 patients (15.0%), with a 93.8% cure rate (30/32). LSR "persisted" in 22 patients (10.3%), all showing 77.3% complete spasm resolution/cure (17/22). In 16 patients (7.4%), LSR disappeared during the opening phase, reaching 100% cure rate (16/16). LSR disappeared following arachnoid dissection in 40 patients (18.7%), with a 91.1% cure rate (36/40). Finally, LSR disappearance following nerve decompression was observed in 104 patients (48.6%), showing a 78.9% cure rate (82/104). Detailed representation of the patients is tabulated in Table 1 and Fig. 2. In 6 patients, initial disappearance of the LSR took place and then the LSR reappeared until they completely re-disappeared following the decompression phase.

**Table 1:** Showing the distribution of our series according to the timing of disappearance of the LSR

Group	No spreads detected	Post opening	Post arachnoid dissection	Post vascular decompression	Persistent LSR	Total
Patients Number	32	16	40	104	22	214
Male:Female	1:1.5	1:1.3	1:1.7	1:1.7	1:1	1:1.6
Mean age (SD)	57.1 (11.3)	47 (10)	55 (12.3)	55.2 (11.5)	54.3 (11.2)	54.9 (11.6)
Disease Duration (SD) years	6.9 (5.1)	8.5 (4.8)	7.9 (5.5)	8.3 (5.9)	7.3 (6.1)	7.9 (5.6)
Follow-up(SD) months	30.3 (11.3)	23.4 (19.8)	24.4 (16.9)	23.4 (15.1)	31.7 (15.5)	25.8 (15.7)
Immediate improvement	29/32 (90.6%)	16/16 (100%)	36/40 (90%)	92/104 (88.5%)	20/22 (90.9%)	193/214 (90.2%)
Long-term Improvement	30/32 (93.8%)	16/16 (100%)	36/40 (90%)	96/104 (92.3%)	19/22 (86.4%)	197/214 (92.1%)
Immediate Favourable outcome	25/32 (78.1%)	15/16 (93.8%)	31/40 (82.1%)	72/104 (69.2%)	18/22 (81.8%)	161/214 (75.2%)
Longterm Favourable Outcome	30/32 (93.75%)	16/16 (100%)	35/40 (91.1%)	82/104 (78.9%)	17/22 (77.3%)	180/214 (84.1%)
Facial Palsy immediate	3/32 (9.4%)	0/16 (0%)	3/40 (7.5%)	9/104 (8.7%)	0/22 (0%)	6/214 (2.8%)
Facial Palsy Longterm	0/32 (0%)	0/16 (0%)	0/40 (0%)	2/104 (1.9%)	0/22 (0%)	2/214 (0.9%)

Comparison of the patient groups was then performed as follows: firstly, the patients in which LSR were detected and then disappeared during the MVD procedure were compared to the patients in which the LSR persisted even after concluding the MVD and closing the skin. The result of this comparison is tabulated in Table 2. Here no statistical significance was noted regarding the outcome and possible confounding variables.

**Table 2:** Comparing patients in which at the end of the operation LSR persisted to patients in which LSR disappeared

Group	LSR disappeared	LSR persisted	P- Value
Number of Patients	160	22	N/A
Male: Female	1:1.7	1:1	0.371364
Mean age (SD) years	54.6 (11.8)	54.3 (11.2)	0.921249
Disease duration (SD) years	8.1(5.7)	7.8 (6.9)	0.807922
Follow-up (SD) months	24.1(15.4)	31.7 (15.5)	0.031685
Immediate Improvemet (%)	144/160 (90%)	21/22 (95.5%)	0.409754
Long-term Improvement (%)	146/160 (91.3%)	19/22 (86.4%)	0.460236
Immediate cure (%)	118/160 (73.8%)	18/22 (81.8%)	0.414238
Long-term cure (%)	132/160 (82.5%)	17/22 (77.3%)	0.55073

N/A stands for not applicable

Additionally, cases in which the LSR were recorded and disappeared before the decompression phase of the MVD (opening phase and arachnoid dissection phase patients) were compared to cases in which LSR were recorded and disappeared following the “decompression phase”. The results for this comparison are tabulated in Table 3. Here it showed only statistical significance regarding the long-term cure rates in which early disappearance of LSR is associated with higher cure rates (p-value < 0.05).

**Table 3:** Comparing the patients regarding the disappearance of the LSR before the actual vascular decompression and after the vascular decompression

Group	Pre Decompression Group	Post Decompression Group	P- Value
Number of Patients	56	104	N/A
Male:Female	22:34 (1:1.5)	38:66 (1:1.7)	0.732074
Mean age (SD) years	53.5 (12.2)	55.2 (11.5)	0.402761
Disease duration (SD) years	8.0 (5.4)	8.2 (5.9)	0.880461
Follow-up (SD) months	25.5 (16.0)	23.4 (15.1)	0.406216
Immediate Improvemet (%)	52/56 (92.9%)	92/104 (88.5%)	0.376701
Long-term Improvement (%)	52/56 (92.9%)	96/104 (92.3%)	0.899845
Immediate cure (%)	46/56 (82.1%)	72/104 (69.2%)	0.07664
Long-term cure (%)	51/56 (91.1%)	82/104 (78.9%)	0.048914*
Facial palsy immediate (%)	3/56 (5.4%)	9/104 (8.7%)	0.450163
Facial palsy long-term (%)	0/56 (0%)	2/104 (1.9%)	> 0.05

N/A stands for not applicable

### Facial weakness and LSR

Facial nerve weakness or palsy is a complication we strive to avoid during MVD for HFS. Since the LSR reflects abnormal electrical firing of the affected facial nerve, we investigated whether the timing of LSR disappearance during the procedural steps correlated with the occurrence of facial nerve weakness. Although a later disappearance of the LSR was associated with a slightly higher incidence of facial palsy, this difference was not statistically significant.

## Case presentation

To further illustrate the possible intraoperative value of LSR, we present a particularly interesting case from our series, where we believe that LSR monitoring played an important role in guiding intraoperative decision-making.

**Fig. 3:**
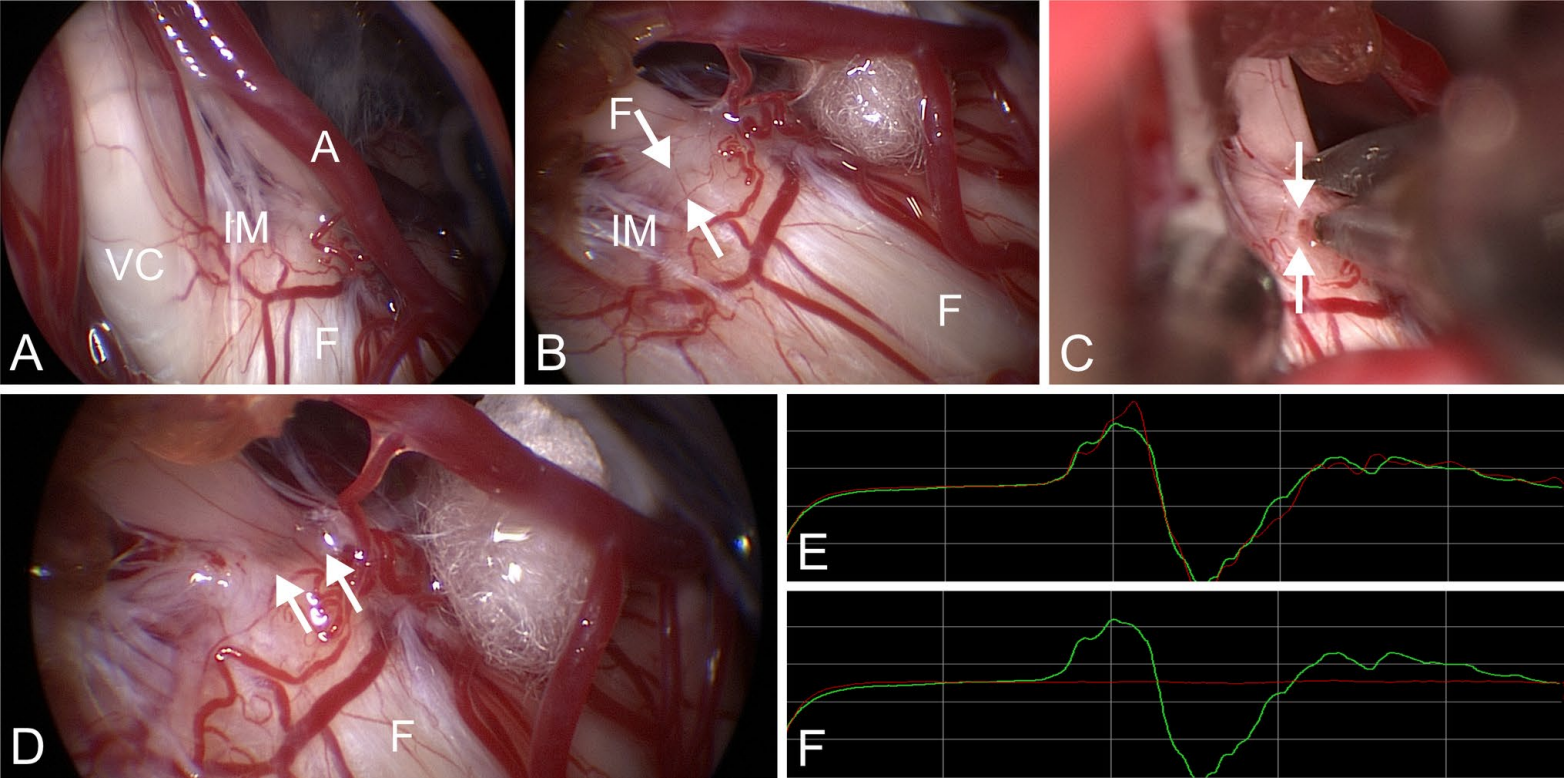
Showing intraoperative images during MVD for hemifacial spasm as well as intraoperative LSR. **A**: Endoscopic image obtained with a 45° endoscope showing the VII-VIII nerve complex (F, VC, IM) and an AICA branch (**A**) vessel in contact with the facial nerve (**F**) as a compressing vessel. **B**: Shows adequate transposition of the vessel using shredded Teflon so that no contact is left between the vessel and the nerve. However, the LSR did not disappear. Close endoscopic inspection showed an arachnoidal stricture (arrows) that might be causing compression of the facial nerve. **C**: Releasing the stricture sharply using scissors. **D**: Shows adequate decompression of the nerve and complete release of the arachnoidal stricture (arrows). **E**: Shows the LSR status following placement of the Teflon pledget with persistence of the LSR. The green curve shows the LSR before starting the operation and the red curve shows the LSR following Teflon pledget placement **F**: Shows complete resolution of the LSR following cutting of the arachnoidal stricture represented by the flat red curve in comparison to the preoperative green curve. (A – anterior inferior cerebellar artery, VC – vestibulocochlear nerve, F- facial nerve, IM – intermediate nerve, arrows – arachnoid stricture)

The patient was a 26-year-old male with a four-year history of HFS (Fig. 3). Intraoperatively, the compressing vessel was identified as shown in Fig. 3A. After adequate decompression using shredded Teflon, as shown in Fig. 3B, LSR remained prominently present (Fig. 3E). This prompted us to investigate further for possible missed compression and upon further exploration with the 45° endoscope, we identified an arachnoid stricture at the root exit zone (REZ) of the nerve (Fig. 3B) which was sharply dissected (Fig. 3C). Following the release of the stricture, the nerve appeared completely free (Fig. 3D) and the LSR disappeared completely (Fig. 3F).

## Discussion

### Main annotations of the study

The primary finding of this study is that the timing of LSR disappearance during MVD for HFS may be associated with long-term surgical outcomes, particularly regarding the cure rate and complete resolution of spasms. In contrast, disappearance of LSR as such was not correlated with long- or short-term results.

### Timing matters

A statistically significant difference regarding the surgical outcome was observed between patients in whom the LSR disappeared before the actual intraoperative decompression of the offending vessel and those in whom LSR disappearance preceded the actual compression and was relieved following minimal surgical maneuvers, such as craniotomy, dural opening, cerebrospinal fluid (CSF) release, and arachnoid dissection. However, the disappearance of LSR as such was not found to be statistically significant with respect to either short- or long-term outcomes.

In approximately 49% of our cohort, the disappearance of the LSR occurred only after the actual intraoperative vascular decompression of the offending vessel took place. This group demonstrated a long-term cure rate and spasm resolution of 78.9% compared to a long-term cure rate of 91.1% in cases with earlier LSR resolution.

Further subgroup analysis reveals that in the smallest group, where the LSR disappeared very early in the procedure—following dural opening and cerebrospinal fluid (CSF) release from the cistern alone (7% of cases)—complete spasm resolution was achieved in all patients (100%).

This may suggest that in cases of presumably milder neurovascular compressions, minimal decompression through dural opening, CSF release, and arachnoid dissection may be sufficient to abolish the LSR. The better recovery observed in this group might be attributed to the possibly assumed lower severity of the initial compression. These findings are consistent with those reported by Jiang et al., who observed early LSR disappearance in approximately 9% of their series before the actual vessel decompression was performed. They attributed this phenomenon to factors such as CSF dynamic changes upon dural opening, CSF diversion, or slight cerebellar retraction. Notably, these patients also experienced better outcomes [7, 15]. Possibly as we assumed, the early loss of the lateral spread response is simply reflecting a less severe form of hemifacial spasm that is more likely to have a good outcome.

However, the absence of a statistically significant association between LSR disappearance as such and MVD outcomes still agrees with our previous studies as well as reported by many other authors in the literature [6, 8, 19].

### LSR disappearance and spasms resolution

Many studies have investigated the prognostic significance of the LSR disappearance vs. persistence on the long-term results of MVD for HFS. Most of these studies concluded that there was no significance as a prognostic factor. However, few studies claimed significance in the short-term but not in the long-term [5, 17, 20]. In a previous study from our series, we evaluated the LSR among 100 patients in our series and there was no significance detected regarding disappearance of the LSR and the postoperative outcome [8]. We repeated this analysis for the current cohort in this study, comparing cases with intraoperative LSR disappearance (n = 160) to those with persistent LSR (n = 22). No statistically significant differences were observed in either short- or long-term outcomes between the two groups.

Similarly, authors have also described no significance of the LSR on the postoperative outcome and only declared its intraoperative helpfulness in most of the cases to guide the microvascular decompression as a tool but without statistical significance on the outcomes. However, this means that in cases where intraoperatively the LSR persists, it does not necessarily reflect a bad outcome and vice versa [6, 19]. A Metaanalysis from 2020 including 26 studies with 7479 patients declared that the final intraoperative LSR status predicted the clinical outcome of MVD with a specificity of 89% and sensitivity of 40% at 1 year and suggested that for adequate MVD, extensive exploration should be performed before closing in patients where intraoperative LSR persistence [28]. One good example for intraoperative usefulness of LSR status, is the case we previously presented in the results (Fig. 3). Interestingly, some authors also examined the LSR 1 month postoperatively and reported prognostic significance which should be also further investigated [17].

### MVD Navigation without sole LSR reliance

Several studies have reported poorer outcomes in cases of early LSR disappearance, contradicting our findings [18]. This discrepancy could be explained by the possible over-reliance on LSR disappearance as the single indicator of adequate decompression. A meticulous intraoperative exploration for the offending vessel should be performed regardless of early LSR disappearance. We emphasize the importance of adequate visualization of the REZ as the gold standard microscopically or endoscopically, in our experience particularly using a 45°- endoscope, ensuring that there is no nerve contact, even with alloplastic materials such as Teflon or Gortex and not solely relying on the LSR status as it might be in some cases unreliable.

Two primary theories have been proposed to explain the occurrence of the LSR in HFS: the central and peripheral theories. Both theories have received experimental support. The central theory is generally favored due to the frequent immediate disappearance of the LSR following decompression which suggests interruption of ephaptic transmission of abnormal impulses to adjacent facial nerve branches, which are presumed to generate the LSR, whereas on the other hand, patients with the persistence of LSR despite adequate decompression support partially the peripheral theory.

Several studies have addressed the limited reliability of LSR disappearance in confirming adequate decompression. Those articles especially stressed on the relationship of the durable disappearance of LSRs with the frequent delayed cure of the HFS as a probable hyperactive central disease linked to the chronic stimulation the facial nerve REZ [7, 12, 21–24, 31, 32].

Furthermore, it should be noted that, in some cases, compressions may be caused by more than one artery, a vein, or even arachnoid bands [11, 33]. Therefore, optimal MVD under proper visualization, combined with the disappearance of the LSR, which may serve as a strong positive indicator of successful decompression.

Conversely, if decompression appears visually adequate but the LSR persists, further exploration for possibly missed compressing vessels is recommended. Nonetheless, even in cases where the LSR persists, more than 77% of patients still experienced positive surgical outcomes, indicating that successful decompression and patient recovery may still occur despite persistent LSR [28]. Interestingly, Helal et al. reported a significant correlation between ongoing oral medical treatment at the time of surgery and the persistence of LSR intraoperatively, despite successful vascular decompression. This finding suggests that patients receiving neuromodulatory agents at the time of MVD are more likely to exhibit persistent LSR at the conclusion of surgery, irrespective of the eventual clinical outcome of HFS [13].

In approximately 15% of cases in our series, no LSR was detectable at the onset of the surgery. We believe this absence could be attributed to various factors. In 20 of the 32 patients without detectable LSR, recent administration of Botulinum toxin (Botox) within three months of surgery was identified as a potential contributing factor. In the remaining 12 patients, the reason for the absence of LSR could not be clearly determined. It might be related to difference in the etiology of the HFS (central vs peripheral theory) or the use of muscle relaxing agents, however our anesthesia protocol for MVD does not include muscle relaxants during the procedure, minimizing this as a likely cause [8, 9].

Despite the absence of LSR as an intraoperative guide in these cases, a cure rate of 93.8% for spasms could be achieved. This suggests that optimal visualization of the surgical field and the surgeon’s experience remain the primary determinants for assessing the adequacy of MVD, even when electrophysiological monitoring is inconclusive.

### Limitations and further studies

Despite the prospective design and relatively large overall sample size (214 cases), our study has several limitations. Notably, the number of patients in specific subgroups—such as the opening phase group (n = 16)—was limited, which may restrict the statistical power and generalizability of findings within these categories. Larger, multicenter studies with balanced subgroup representation are needed to confirm and expand upon our observations.

Additionally, our study did not assess long-term outcomes in correlation with LSR disappearance at predefined “postoperative intervals” and only assessed it intraoperatively. This aspect has been explored by other investigators [17]. Incorporating such follow-up assessments in future research could provide a more comprehensive understanding of the prognostic value of intraoperative LSR changes. Furthermore, the potential clinical relevance of intraoperative amplitude and latency fluctuations of LSR was not examined and warrants investigation in future studies.

## Conclusions

Although LSR status may not significantly influence postoperative MVD outcomes, it may serve as a useful intraoperative adjunct during MVD for HFS, particularly when combined with optimal visualization and surgical expertise. Early disappearance of the LSR during the initial stages of decompression may indicate favorable long-term outcomes. Nonetheless, the majority of patients undergoing MVD for HFS experience substantial symptomatic improvement, irrespective of intraoperative LSR findings.

## Data Availability

No datasets were generated or analysed during the current study.
